# Cytokine Profiles Associated With Worse Prognosis in a Hospitalized Peruvian COVID-19 Cohort

**DOI:** 10.3389/fimmu.2021.700921

**Published:** 2021-09-01

**Authors:** Maria J. Pons, Barbara Ymaña, Ana Mayanga-Herrera, Yolanda Sáenz, Lydia Alvarez-Erviti, Salyoc Tapia-Rojas, Roxana Gamarra, Amanda B. Blanco, Gemma Moncunill, Manuel F. Ugarte-Gil

**Affiliations:** ^1^Grupo Enfermedades Emergentes, Universidad Científica del Sur, Lima, Peru; ^2^Laboratorio de Cultivo Celular e Inmunología, Universidad Científica del Sur, Lima, Peru; ^3^Área de Microbiología Molecular, Centro de Investigación Biomédica de La Rioja (CIBIR), Logroño, Spain; ^4^Área de Neurobiología Molecular, Centro de Investigación Biomédica de La Rioja (CIBIR), Logroño, Spain; ^5^Hospital Nacional Guillermo Almenara Irigoyen, EsSalud, Lima, Peru; ^6^ISGlobal, Hospital Clínic - Universitat de Barcelona, Barcelona, Spain

**Keywords:** COVID-19, cytokine, prognosis, SARS-CoV-2 IgG, proinflammatory, biomarkers

## Abstract

Cytokines, chemokines and growth factors present different expression profiles related to the prognosis of COVID-19. We analyzed clinical parameters and assessed the expression of these biomarkers in patients with different disease severity in a hospitalized Peruvian cohort to determine those associated with worse prognosis. We measured anti-spike IgG antibodies by ELISA and 30 cytokines by quantitative suspension array technology in 123 sera samples. We analyzed differences between patients with moderate, severe and fatal COVID-19 by logistic regression at baseline and in longitudinal samples. Significant differences were found among the clinical parameters: hemoglobin, neutrophils, lymphocytes and C-reactive protein (CRP), creatinine and D-dimer levels. Higher anti-spike IgG antibody concentrations were associated to fatal patient outcomes. At hospitalization, IL-10, IL-6, MIP-1α, GM-CSF, MCP-1, IL-15, IL-5, IL1RA, TNFα and IL-8 levels were already increased in fatal patients´ group. Meanwhile, multivariable analysis revealed that increased GM-CSF, MCP-1, IL-15, and IL-8 values were associated with fatal outcomes. Moreover, longitudinal analysis identified IL-6 and MCP-1 as the main risk factors related to mortality in hospitalized COVID-19 patients. In this Peruvian cohort we identified and validated biomarkers related to COVID-19 outcomes. Further studies are needed to identify novel criteria for stratification of SARS-CoV-2 infected patients at hospital entry.

**Background:**

In the most severe forms of SARS-CoV-2 infection, large numbers of innate and adaptive immune cells become activated and begin to produce pro-inflammatory cytokines, establishing an exacerbated feedback loop of inflammation.

**Methods:**

A total of 55 patients with laboratory-confirmed COVID-19 admitted to the *Hospital Nacional Guillermo Almenara Irigoyen* in Lima, Peru were enrolled during August-October 2020. Of these, 21 had moderate disease, 24 severe diseases and 10 died. We measured 30 cytokines and chemokines by quantitative suspension array technology and anti-spike IgG antibodies using a commercial ELISA. We evaluated these parameters in peripheral blood every 2-5 days until patient discharge or death. Patient information and clinical parameters related were obtained from the respective clinical histories.

**Results:**

The frequency of obesity differed among the 3 groups, being most frequent in patients who died. There were also significant differences in clinical parameters: hemoglobin, segmented neutrophils, lymphocytes,C-reactive protein, creatinine and D-dimer levels. Greater anti-spike IgG antibody concentrations were associated to fatal outcomes. In univariate analyses, higher baseline concentrations of IL-6, MIP-1α, GM-CSF, MCP-1, IL-15, IL-5, IL1RA, TNFα, IL-8 and IL-12p70 correlated with severity, while multivariable analysis showed that increased concentrations in 4 biomarkers (GM-CSF, MCP-1, IL-15, IL-8) were associated with fatal outcomes. Longitudinal analysis showed IL-6 (hazard ratio [HR] 6.81, 95% confidence interval [CI] 1.6-28.7) and MCP-1 (HR 4.61, 95%CI 1.1-19.1) to be related to mortality in hospitalized COVID-19 patients.

**Conclusions:**

Cytokine, chemokine and growth factor profiles were identified and validated related to severity and outcomes of COVID-19. Our findings may be useful to identify novel criteria for COVID-19 patient stratification at hospital entry.

## Introduction

The severe acute respiratory syndrome-related coronavirus (SARS-CoV-2), which emerged in December 2019 in China, is highly pathogenic and is one of the greatest threats to public health worldwide (1). The effects of SARS-CoV-2 in infected patients are variable, with around 20% requiring hospital admission, and of these, 5% requiring ventilatory support in an intensive care unit (ICU) (2). The most serious presentations are associated with systemic inflammation (3).

The pathophysiology of COVID-19 is still under study and further research is needed to assess the causes and biomarkers of the highly variable degrees of disease progression (4). It has been described that the acute inflammatory response observed in patients with severe disease is related to an increase of some inflammatory markers such as C-reactive protein (CRP), ferritin, D-dimer, neutrophil-to-lymphocyte ratio. In addition, elevated levels of some inflammatory cytokines and chemokines have been observed in patients with severe diseases (5–8).

It is necessary to delineate the role of cytokines, chemokines and growth factors in the pathogenesis of COVID-19 across the spectrum of disease severity in different populations, ages and with and without comorbidities. Identifying prognostic biomarkers will allow prioritization of hospital resources and personalized treatment. Hence, there is great interest in determining how the severe acute respiratory syndrome alters the normal antiviral immune responses and biomarkers associated (9, 10). Our study aimed to identify immune-based biomarkers associated with clinical outcomes in patients hospitalized with different degrees of COVID-19 severity.

## Material and Methods

### Study Population

A cohort study was carried out including patients diagnosed with moderate or severe COVID-19 (by PCR or antibodies) hospitalized at the *Hospital Nacional Guillermo Almenara Irigoyen*. Patients were recruited at the time of admission to the hospital, during the period from August to October 2020. This period (mainly, August-September) coincided with the peak of active cases of the first wave of COVID-19 in Peru. It should be noted that the capacity of the available health resources, both material and human, was overstretched.

### Definition of Disease Severity

Patients were stratified as having moderate or severe infection. Moderate infection was defined as requiring hospitalization and presenting an arterial oxygen partial pressure (*PaO2* in mmHg) to fractional inspired oxygen (Pa/FiO_2_) ratio greater than 100 or pulse oximetric saturation Sa/FiO_2_ greater than 89 and Pa/FiO_2_ less than 200 or Sa/FiO_2_ less than 214 according to the Berlin 2012 definition of ARDS, and severe infection was defined as patients with at least one of the following conditions: having a Pa/FiO_2_ ratio less than 100 or Sa/FiO_2_ less than 89; respiratory failure requiring mechanical ventilation; shock or other organ failure requiring ICU admission.

### Clinical Data

From the medical records we collected data related to age, gender, date of hospitalization, date of progression to severity and date of discharge or death, comorbidities (hypertension, coronary disease, chronic respiratory disease, diabetes, obesity and any other chronic underlying disease) as well as the pharmacological treatment (corticoids) received.

### Samples

Following serological tests processed within maximum 2 hours, a total of 123 serum samples were collected (BD Vacuum Tube Vacutainer Gel SST 5 ml) and kept at -20°C in several aliquots in the Microbiology Laboratory of the *Hospital Nacional Guillermo Almenara Irigoyen.* The samples were then transferred as category B biological (triple packaging) to the laboratories of the *Universidad Científica del Sur* and stored at -80°C and thawed only once prior to multiplex analysis.

### Quantification of Cytokines, Chemokines, and Growth Factors

Biomarker analyses were performed using Luminex and a commercial human cytokine magnetic bead kit (Milliplex Human Cytokine/Chemokine MAGNETIC BEAD Premixed 30 Plex Kit). This kit measures the concentrations (pg/mL) of the following cytokines, chemokines and growth factors: epidermal growth factor (EGF), eotaxin (CCL11), granulocyte colony stimulating factor (G-CSF), granulocyte macrophage colony stimulating factor (GM-CSF), interferon (IFN)-α, IFN-γ, interleukin (IL) -1α, 1RA, IL-1β, IL-2, IL-12, IL-3, IL-4, IL-5, IL6, IL-7, IL-8, IL-10, IL-12 (p40/p70), IL-13, IL-15, IL-17, IP-10, monocyte chemoattractant protein-1 (MCP-1), macrophage inflammatory protein (MIP)-1α, MIP-1β, RANTES, tumor necrosis factor (TNF α), TNF β and vascular endothelial growth factor (VEGF). Fifty µl of all the serum samples were tested according to the manufacturer’s instructions in individual replicates distributed on two plates. Each plate included 8 dilutions of a standard for standard curves provided by the vendor with a known concentration of each analyte. Two blank controls and three positive controls in duplicates of high, medium and low concentrations prepared from a reference sample were also included on each plate for quality control and quality assurance purposes. The samples were measured using MAGPIX^®^ and the results were analyzed with xPONENT 3.1 software.

### Measurement of Antibodies Anti-SARS-CoV-2

Anti-spike SARS-Cov-2 protein IgG was evaluated by a semi-quantitative commercial kit (EuroImmune), according to manufacturer’s instructions. The results were evaluated by calculating a ratio of the absorbance of the patient sample to the absorbance of the calibrator (>1 results were considered positive). The cut-off was defined following the manufacturer’s recommendations. The absorbance values were read on the Biotek Synergy LX Multi-Mode Reader (Agilent).

### Statistical Analysis

We performed a descriptive analysis summarizing each categorical variable with the absolute and relative frequencies and each numerical variable with the mean and standard deviation. We normalized cytokine concentration with its logarithm with base 10. Then, we compared each descriptor by outcome (moderate, severe, and death) using the chi square test for proportion comparisons and the Kruskal-Wallis test with Dunn´s test for levels comparisons. Correlations between clinical parameters, immunoglobulin levels, and cytokine, chemokine and growth factor concentrations were assessed by Spearman tests. To identify clusters of biomarkers associated with the severity group, we performed Parial Least Squares - Discriminant Analysis *(PLS-DA*). The analyses of prognostic factors were performed using logistic regression. We performed two sets of analysis. First, we assessed the predictability of death using only the patient descriptors at baseline. This analysis included a Spearman correlation analysis and regression analysis using generalized linear models with a Poisson distribution, link log, and robust error variance. Second, we performed longitudinal and association analysis between biomarkers with mortality using Cox regression models. Hazard ratios (HR) were therefore estimated as the magnitude of the association of interest. All models were fitted using the forward method and the Akaike Information Criterion. Each parameter was estimated using a confidence interval of 95% (95% CI). All the analyses were performed using STATA MP version 14.0 (Stata Corp., College Station, TX) and all the graphics were elaborated using R studio version R-4.0.2.

### Ethical Aspects

This project was approved by the Institutional Ethics Committee of the *Universidad Científica del Sur* and the Research Ethics Committee for COVID-19 of IETSI-EsSalud (42-IETSI-ESSALUD-2020).

In order to avoid exposing workers to infectious areas and the need to obtain additional samples from patients, leftover serum samples taken for diagnostic purposes during hospitalization were analyzed. No additional samples were taken from the patient, so no additional inconvenience was generated.

## Results

### Baseline Clinical Characteristics of COVID-19 Hospitalized Patients

Fifty-five patients hospitalized for COVID-19 disease were included: 21 (38.2%) in the moderate disease group, 24 (43.6%) in the severe group and 10 in the deceased group (18.2%), according to maximum state category reached during the follow-up period. The mean age of the patients was 54, 51 and 52 years, respectively, with no differences in the median age and sex among the 3 groups (p=0.360, p=0.244, respectively). Regarding comorbidities, 24 patients (43.6%) had no comorbidities and 31 (56.4%) had at least one comorbidity, including 15 (27.3%) patients with obesity, 12 (21.8%) with hypertension, and 7 (12.7%) with diabetes. Only obesity presented differences between severity groups, being more frequent in the deceased group (p=0.002) (**Table 1**). Moreover, two patients (3.6%) had obesity, hypertension, and diabetes and two (3.6%) patients had both obesity and hypertension. Finally, only 1 patient (1.9%) had hypertension and diabetes and 1 patient (1.9%) presented obesity and diabetes. The presence of other comorbidities such as chronic kidney disease, chronic obstructive pulmonary disease (COPD), cardiovascular disease (CVD) and gout is shown in **Table 1**. No patients with liver disease or deep vein thrombosis (DVT) were reported. All hospitalized patients received corticosteroid treatment from the beginning of their stay.

**Table 1:** Characteristics of the study population.

	Moderate	Severe	Deaths	p-value
Overall, (n,%)	21 (38.2)	24 (43.6)	10 (18.2)	
Men (n,%)	17 (80.9)	22 (91.7)	10 (100)	0.244
Age, mean (sd), yr	54.6 (16)	51.1 (11.2)	52 (14.3)	0.360
Hospital discharge, mean (sd), days	11.9 (8.2)	12.9 (7.1)	13.5 (7.1)	0.898
Comorbidities				
None, n (%)	11 (52.4)	12 (50.0)	1 (10)	0.059
Obesity, n (%)	2 (9.5)	6 (25.0)	7 (70)	**0.002**
Diabetes, n (%)	4 (19.1)	1 (4.2)	2 (20.5)	0.245
Hypertension, n (%)	4 (19.1)	5 (20.8)	3 (30)	0.779
CKD, n (%)	0 (0)	1 (4.2)	0 (0)	0.518
COPD, n (%)	1 (4.8)	0 (0)	2 (20)	0.064
CVD, n (%)	0 (0)	0 (0)	1 (10)	0.101
DVT, n (%)	0 (0)	0 (0)	0 (0)	NA
Gout, n (%)	1 (4.8)	0 (0)	0 (0)	0.438
Other diseases	4 (19.1)	1 (4.2)	0 (0)	0.121
Hemogram				
Hemoglobin, mean (sd), g/dL	14.3 (1.7)	14.6 (1.4)	12.7 (1.2)	**0.004**
Leukocytes, mean (sd), 10^9^/L	10.4 (4.6)	10.6 (3.9)	13.1 (3.0)	0.208
Basophiles, mean (sd)	2.8 (3.4)	1.9 (1.5)	2 (1.5)	0.602
Segmented neutrophils, mean (sd)	81.1 (12.0)	81.2(6.2)	89.3 (4.6)	**0.037**
Lymphocytes, mean (sd)	13.3 (10.7)	12.5 (5.7)	6.3 (4.1)	**0.001**
Neutrophils, mean (sd), (total)	8.9 (4.9)	8.8 (3.6)	11.8 (3.1)	0.124
lymphocytes, mean (sd), (total)	1.1 (0.7)	1.2 (0.5)	0.8 (0.5)	0.146
CRP, mean (sd), mg/ml	125 (109)	57 (67)	179 (121)	**0.007**
Platelets, mean (sd), count/ul	3336857 (94290)	363000 (113701)	274750 (41709)	0.101
Creatinine, mean (sd), mg/dl	0.8 (0.6)	0.8 (0.5)	1.8 (1.9)	**0.024**
D-Dimer, mean (sd), ug/ml	0.9 (0.7)	0.8 (0.9)	3.0 (1.6)	**<0.001**
Prothrombin Time, mean (sd), sec.	11.2 (0.8)	11.3 (0.8)	10.8 (0.5)	0.347
TTPA, mean (sd), sec.	32.2 (4.3)	31.3 (4.8)	34.8 (6.4)	0.309
SaFiO_2_, mean (sd)	242.7 (98.1)	240.1 (100.6)	155.7 (79.2)	**0.047**
Ferritin, mean (sd), ug/L	759.1 (389)	968.3 (630)	1247 (754)	0.103

CKD, Chronic kidney disease; COPD, Chronic obstructive pulmonary disease; CVD, Cardiovascular disease; DVT, Deep vein thrombosis; CRP, C-reactive protein; TTPA, activated Partial Thromboplastin Time; P-value < 0.05 were statistical significance and values are marked with bold numbers.

### Clinical Factors and Laboratory Tests at Baseline Associated With Differential Mortality

Regarding hematological parameters, there were significant differences in hemoglobin levels at baseline, being lower in the deceased group (12.7; ± 1.2) (p=0.004) (**Table 1**). In addition, lymphocyte levels were also lower in the severe and deceased groups (p=0.001), while the more severe groups presented higher mean segmented neutrophil values (p=0.037). No significant differences were observed between the 3 groups for the remaining hematological parameters.

With respect to analytical parameters, significant differences were observed in CRP, D-dimer and creatinine values between the moderate, severe and deceased groups (p=0.007, p<0.001 and 0.020, respectively), with the deceased group presenting the highest mean values. There were differences between groups in relation to the Sa/FiO_2_ ratio, with the deceased group also showing lower values than the other two groups (**Table 1**).

### Anti-Spike IgG Antibodies of SARS-CoV-2 at Baseline

**Figure 1:**
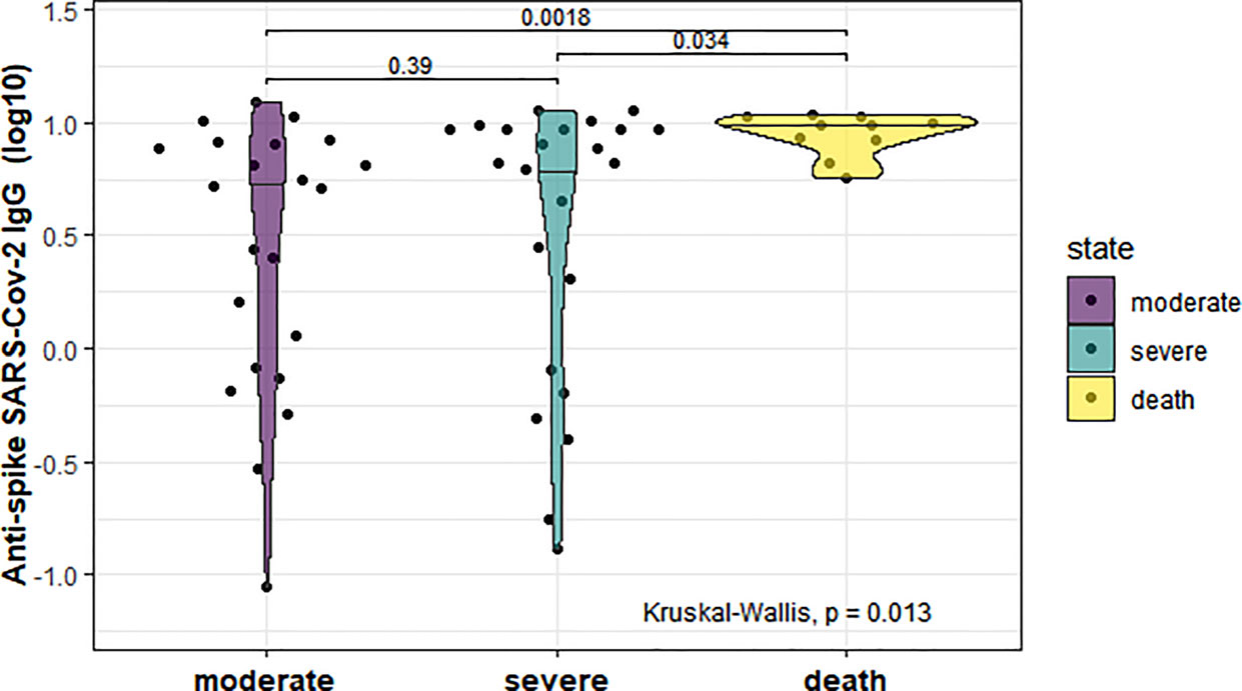
Serum anti-spike IgG concentrations in the three study groups (moderate, severe and deceased) of hospitalized COVID-19 patients at baseline. The median is shown in different violin groups of biomarkers. P-values were computed by the Kruskal-Wallis test and a post-hoc analysis by Dunn’s test.

Most patients had detectable antibodies against the spike 1 protein of SARS-CoV-2, except for 7 patients (12.7%). As shown in **Figure 1**, there were significant differences among the 3 groups (p=0.013), with higher levels observed in the deceased group compared to the moderate and severe group.

### Cytokine Levels in the Moderate, Severe, and Deceased Groups at Baseline

**Figure 2:**
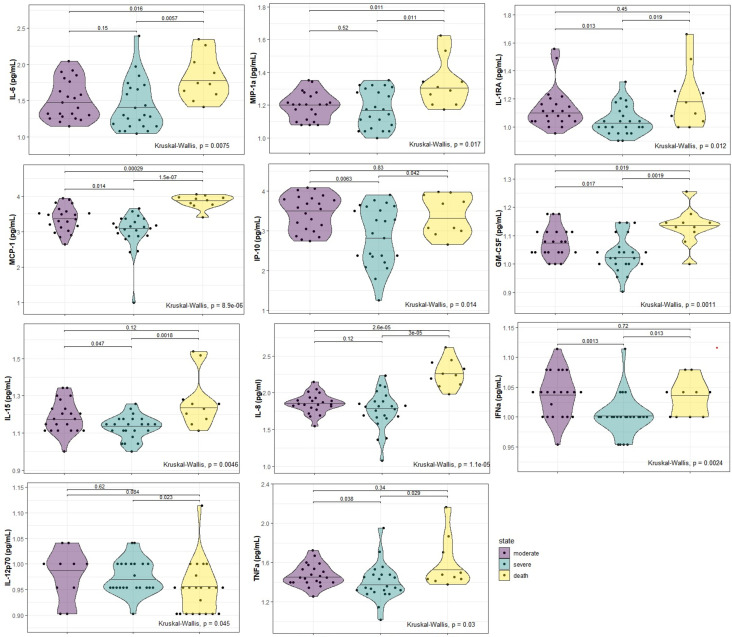
Serum biomarkers with significant differences in the three study groups (moderate, severe and deceased) of hospitalized COVID-19 patients at baseline. P-values were computed by the Kruskal-Wallis test and a post-hoc analysis by Dunn’s test.

Significant differences were found between groups for, IL-6 (p=0.0075), MIP-1α (p=0.017), GM-CSF (p=0.001), MCP-1 (p<0.001), IL-15 (p=0.005), IFNα (p=0.002), IL-1RA (p=0.012), TNFα (p=0.030), IP-10 (p=0.014), IL-8 (p<0.001) and IL-12p70 (p=0.045) levels. All the biomarkers presented higher values in the deceased group than the moderate and/or severe group (**Figure 2**).

### Cytokine and Antibodies Correlations at Baseline

**Figure 3:**
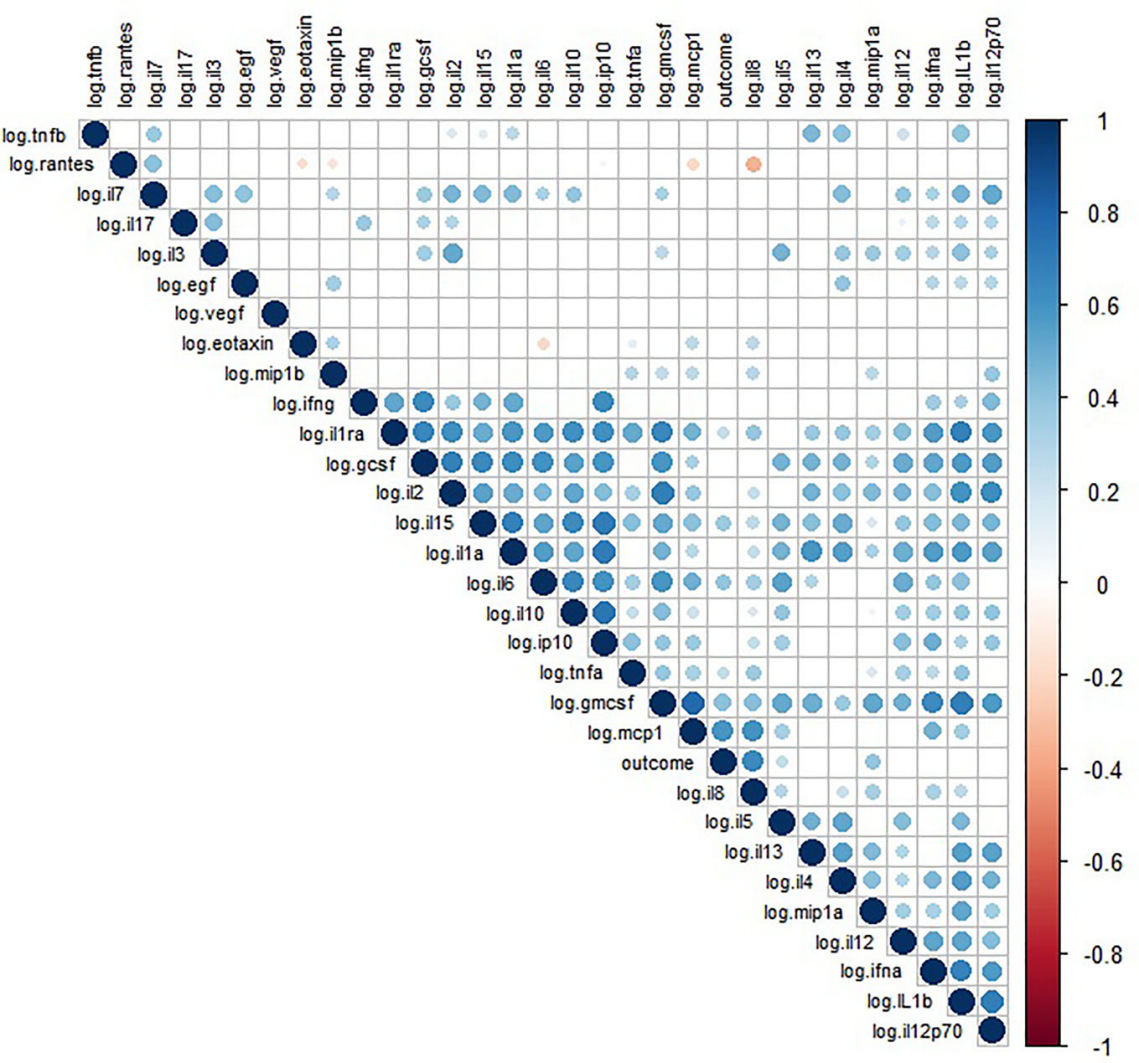
Spearman correlation matrix between biomarkers concentrations. Only significant correlations are shown (p > 0.05). The outcome in this analysis is death.

We observed a significantly high correlation between biomarker levels in COVID-19 patients (**Figure 3**). Positive correlations were found between pro-inflammatory and anti-inflammatory biomarkers, while negative correlations were only observed for RANTES and IL-8. The biomarkers EGF, VEGF, eotaxin, MIP-1β, and TNF-β did not show any significant correlations with the other biomarkers.

**Figure 4:**
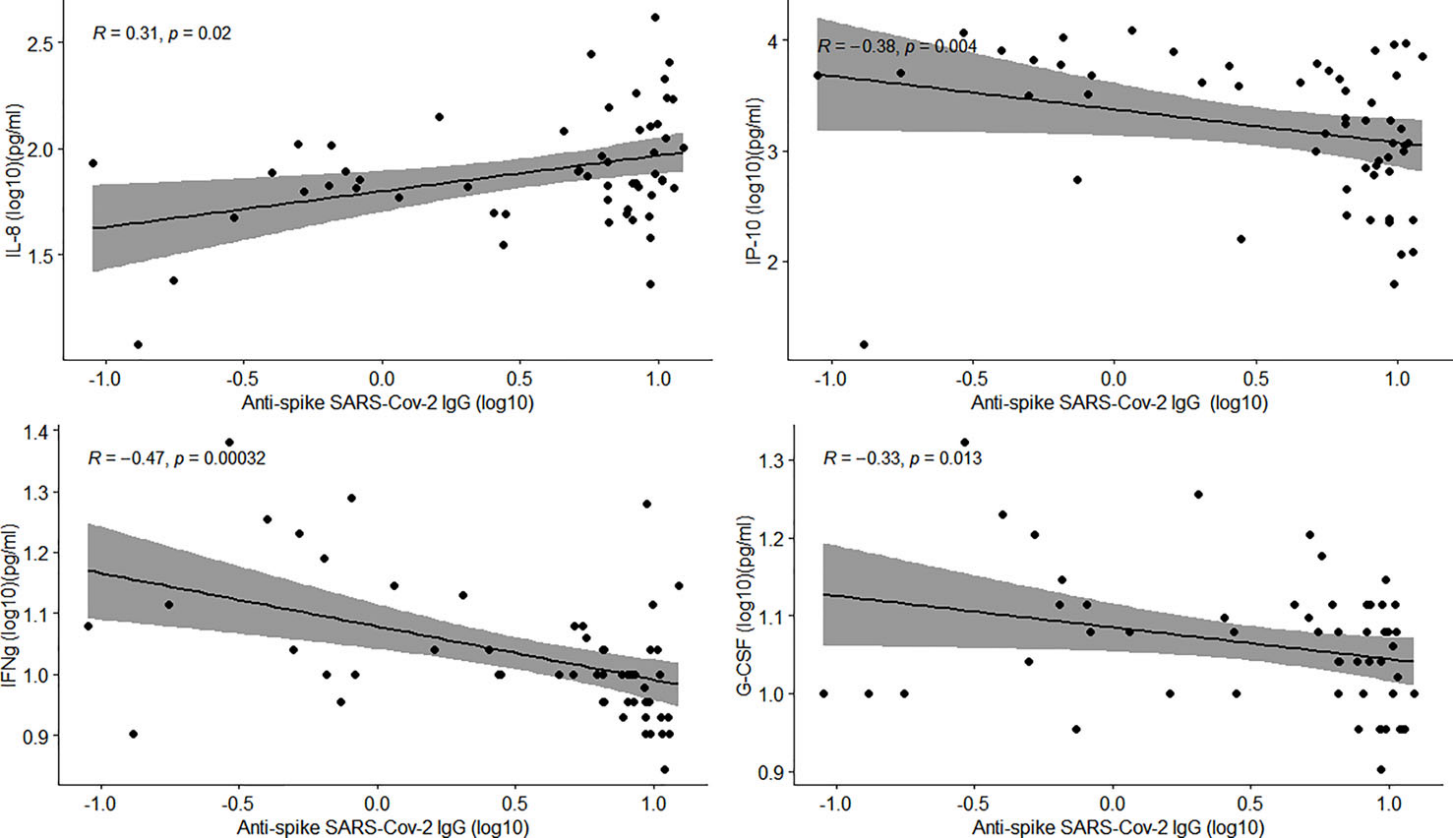
Correlation of anti-spike IgG antibody levels and biomarker concentrations in COVID-19 patients. Scatter plots show statistically significant correlations between biomarkers and antibody levels. Rho value and p-vale were computed by Spearman correlations. The shaded area shows the 95% confidence interval.

There was a positive and moderate correlation between anti-spike IgG and IL-8 (ρ=0.31; p=0.02), while G-CSF (ρ=-0.33; p=0.013), IFN-γ (ρ=-0.47; p<0.001) and IP-10 (ρ=-0.38; p=0.004) showed an inverse correlation (**Figure 4**).

### Multi-Biomarker Associations at Baseline

**Figure 5:**
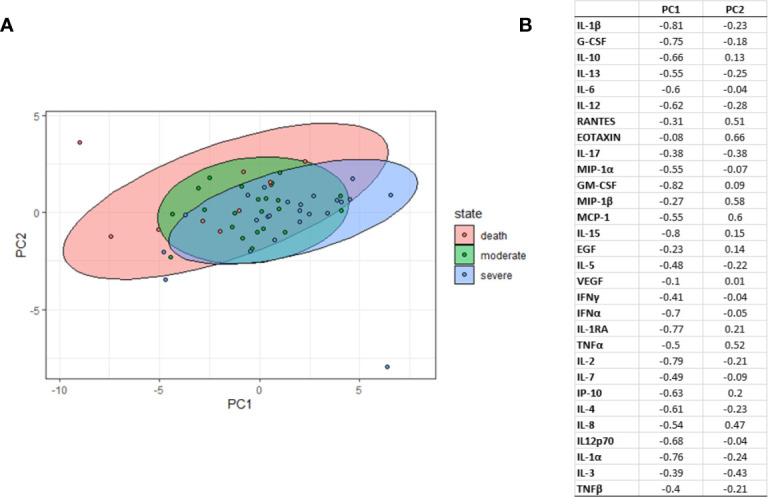
Partial Least-Squares Discriminant Analysis (PLS-DA). **(A)** Graph of PLS-DA plots representing each sample (dots) with respect to the first 2 PLS-DA components according to severity group (moderate, severe, deceased). **(B)** Graph of marker loading to the 2 components of the PLS-DA. Principal component (PC).

In the PLS-DA analysis we identified several components that were negatively associated with the severe COVID-19 group. **Figure 5A** shows the most predictive components (components 1, 2). The biomarkers contributing most to the components were those with higher loadings in the different components (high relevant biomarkers were considered with loadings <-0.50 and very high relevant biomarkers were considered with loadings <-0.75). In component 1, the most relevant biomarkers were IL-10, IL-13, IL-6, IL-12, MIP-1α, MCP-1, IFNα, IL1RA, IP-10, IL-4, IL-8, and IL-12p70 all of which had negative loadings. Additionally, the biomarkers with the highest relevance were IL-1β, G-CSF, GM-CSF, IL-15, IL-2 and IL-1α, also with negative loadings (**Figure 5B**).

### Biomarkers at Baseline Associated With Mortality in Bivariate and Multivariable Regression Analysis

We performed bivariate regression analysis for each of the 30 cytokines, chemokines, growth factors and IgG antibodies to identify those associated with mortality. Increased levels of 11 biomarkers at baseline were associated with increased mortality. These biomarkers included anti-spike IgG levels (IRRc=188.57), biomarkers associated with monocyte/macrophage activation and/or activation of NF-κB MCP-1/CCL2 (IRRc=7.09) and IL-6 (IRRc=2.38), as well as proinflammatory chemokine MIP-1α (CXCL10) (IRRc=10.88) and IL-8, IRRc=7.31 and stimulatory type proinflammatory Th1 cytokines, TNFα (IRRc=2.68) and IL-15 (IRRc=9.99); the Th2 mediator IL-5 IRRc=(5.43), and anti-inflammatory cytokine IL-10 (IRRc=1.61) and IL-1RA (IRRc=3.00) (**Table 2**). The growth factor GM-CSF (IRRc=188.57) was also associated with death (**Table 2**). In the multivariable analysis, the biomarkers GM-CSF (IRRa= 0.003), MCP-1 (IRRa=17.99) and IL-8 (IRRa=3.80) and anti-spike IgG (IRRa=16.34) were significantly associated with mortality (**Table 2**).

**Table 2:** Bivariate and Multivariable regression analysis of biomarkers associated with death in hospitalized COVID-19 male patients.

	Bivariate		Multivariable	
	IRR	95% CI	IRRa	95% CI
GM-CSF	188.57	13.69 – 2597.52	0.003	0.0001 – 0.09
MCP-1	7.09	2.91 – 17.28	17.99	6.81 -47.49
IL-8	7.31	3.75 – 14.25	3.80	1.47 – 9.81
Anti-spike IgG antibody	6.66	1.68 – 26.37	16.340	6.72 – 39.74
MIP1α	10.88	4.56 – 25.94		
IL-15	9.99	4.08 – 24.08		
IL-5	5.43	1.99 – 14.83		
IL-1RA	3.00	1.26 – 7.13		
TNFα	2.68	1.45 – 4.96		
IL-6	2.38	1.46 – 3.88		
IL-10	1.61	1.03 – 2.83		

IRR, Incidence Rate Ratio; IRRa, Incidence Rate Ratio adjusted.

### Longitudinal Analysis and Association Between Biomarkers and Mortality

**Figure 6:**
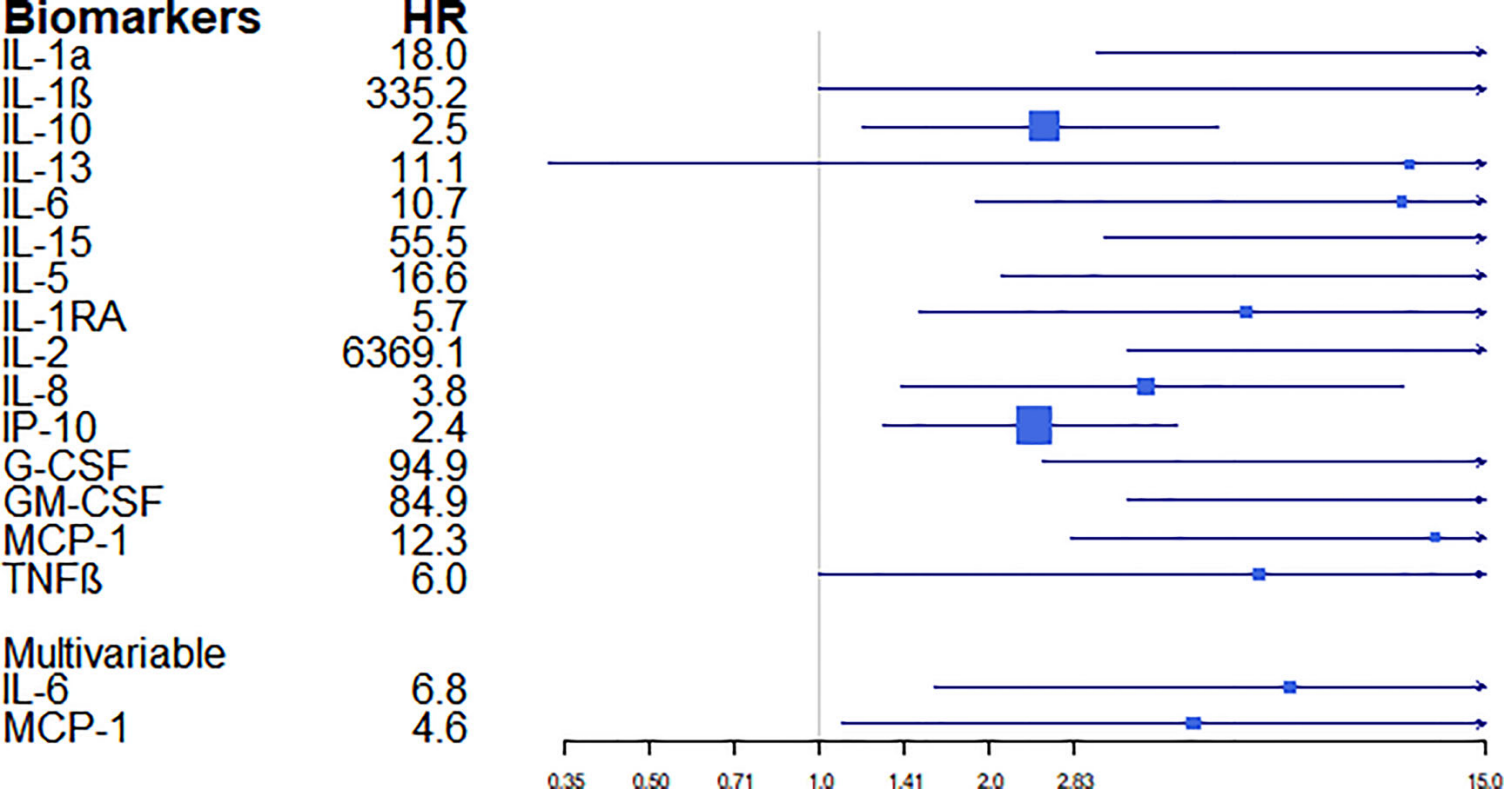
Estimates of the magnitude of association (hazard ratios –HR-) and their confidence intervals between biomarkers and the outcome “death”. HR were computed by univariable and multivariable Cox models.

In the longitudinal bivariate analysis, we observed a significant association between mortality and 15 biomarkers: IL-1α (p=0.001), IL-1β (p=0.050), IL-10 (p=0.012), IL-13 (p=0.007), IL-6 (p<0.001), IL-15 (p<0.001), IL-5 (p=0.008), IL1RA (p=0.010), IL-2 (p=0.022), IL-8 (p=0.007), IP-10 (p=0.004), G-CSF (p=0.015), GM-CSF (p=0.006), MCP-1 (p=0.001), and TNFβ (p=0.045). However, in the Cox regression multivariable analysis the main predictors of death by COVID-19 in hospitalized patients were IL-6 (HR =6.81; 95% CI: 1.6-28.7) and MCP-1 (HR =4.61; 95% CI: 1.1-19.1) (**Figure 6**).

## Discussion

We have profiled the cytokine/chemokine/growth factor and clinical parameters associated with COVID-19 fatal outcome in Peruvian hospitalized patients. Clinical parameters associated with death were lower hemoglobin and lymphocytes, and higher segmented neutrophils, CRP, creatinine and D-dimer levels. Higher concentration of IgG anti-spike-1 antibodies patients were also associated with mortality, as well as higher IL-10, IL-6, MIP-1α, GM-CSF, MCP-1, IL-15, IL-5, IL-1RA, TNFα and IL-8 in bivariable regression analysis and GM-CSF, MCP-1, IL-15, IL-8 in multivariable regression analysis. Longitudinal analysis showed higher concentrations of IL-6 and MCP-1 associated with higher risk of mortality in COVID-19 hospitalized patients. The mean age of 53 years of our study participants was lower than that of other studies in which older age was identified as a predictor of poor outcomes and were mainly composed of men, with male sex also being associated with fatal outcomes (11). Comorbidities have been described as playing an important role in prognosis. We found a high frequency of obesity (70%) in the deceased group. Of note, a significant positive correlation has been reported between COVID-19 infections, mortality and the prevalence of obesity (12). Indeed, previous studies in other Latin cohorts such as a Mexican one (13) have described obesity as the strongest predictor for severe COVID-19, followed by the presence of diabetes and hypertension. However, we found no differences among groups for diabetes and hypertension. This is of relevance given that the prevalence of obesity in Peru is close to 20% (12). Obesity confers chronic inflammation that can modify innate and adaptive immune responses, resulting in a less responsive immune system and greater vulnerability to infection (14).

We found that lymphocyte concentrations were lower in patients with a worse prognosis, being significantly associated with death and suggesting that lymphopenia correlates with the severity of infection. Indeed, it has been proposed that lymphocyte values are a reliable predictor of clinical outcomes in patients with COVID-19, and may be useful to establish prognosis in the clinical management of patients with this disease (15, 16). Furthermore, differences in CRP, D-dimer and creatinine levels had been previously described among groups with different severity, with increased D-dimer levels being associated with worse clinical outcomes, and increased CRP levels with disease progression in COVID-19 patients (5, 17).

Cytokine and chemokine levels can considerably vary during the course of COVID-19 based on the phase of the disease, the administration of immunomodulatory medications or the intrinsic characteristics of the patients (18). We found that at baseline and during hospitalization, some immune markers were predictive of subsequent clinical progression, and could, therefore, identify patients at risk of death. In our analysis we observed that several biomarkers, including GM-CSF, MCP-1, MIP1α, TNFα, IL-1RA, IL-6, IL-8, IL-15 and IL-10 were associated with fatal outcomes in COVID-19. However, our multivariable analysis showed GM-CSF, MCP-1, and IL-15, IL-8 as independent biomarkers associated with fatal outcomes.

The finding of markedly increased monocyte/macrophage activation markers such as MIP-1α and MCP-1 in COVID-19 patients is similar to a previous study describing elevated MIP-1α levels in all COVID-19 patients and elevated MCP-1 values in patients who died (19). In addition, IP-10 and MCP-3 levels have been shown to be highly associated with disease severity and predict the progression (20). The NF-κB dependent proinflammatory biomarkers, IL-6 and TNFα, were increased in both the severe and deceased patient groups, similarly to previous reports (19, 20). Both cytokines were also described in response to spike protein stimulation in SARS-coronavirus (21).

Early and persistent expression of proinflammatory cytokines culminates in systemic hyperinflammation associated with the sudden development of respiratory failure requiring hospitalization and often admission to the ICU (7). Regarding IL-6, some reports have only found a significant association in later stages of the disease and mortality (22, 23). However, in our study, significantly higher levels were reported from hospital admission, and were predictive of disease progression. Despite the association of these biomarkers in patients with greater severity, higher IL-6 and IL-8 levels were also observed with non-COVID-ARDS (24). IL-8 is considered an important chemotactic factor, involving neutrophil activation and recruitment, associated with COVID-19 prognosis, and has previously been proposed as a biomarker of ARDS (25). Moreover, IL-8 activates neutrophils as demonstrated by higher counts in deceased COVID-19 patients. Similar to IL-8, we found IL-15 at baseline and longitudinally to be associated with mortality. IL-15 is involved in inflammatory responses, promoting the production and secretion of IL-8 by neutrophils, which contribute to migration and recruitment at inflammatory sites (26). Furthermore, IL-10 amplifies viral sepsis-related hyperinflammation in critically ill and severe COVID-19 patients and is linked to T-cell exhaustion, presumably through overactivation and proliferation (27). Previously, higher expression of pro-inflammatory cytokine levels (IFN-α, TNF, IL-6, IL-8, IL-10, and IP-10) were described in monocyte-derived macrophages in SARS-CoV infections (28). Among these, IP-10, IL-10 and IL-6 have been proposed as a triad predictive of subsequent COVID-19 clinical progression (5).

In addition, IL-6 and MCP-1 have also been proposed as candidate markers for disease prediction in hospitalized patients with respiratory failure (29), and in the regression analysis we found IL-6 and MCP-1 in hospitalized patients to be the main predictors of death. MCP-1 levels suggest that secretion is associated with lung injury in severe COVID-19 (30) playing a pathogenic role in respiratory failure during hospitalization (29).

In relation to growth factors, we found that G-CSF and GM-CSF levels were related to disease severity. These biomarkers are associated with inflammatory conditions and promote cytokine production in macrophages in the presence of inflammation, perpetuating the inflammatory process in patients with a poor prognosis (31). In our study in both baseline and longitudinal samples, GM-CSF was identified as a biomarker of fatal outcome, although in a previous study no significant differences in severe and moderate disease groups were described (22).

Furthermore, IL-1RA, a negative regulator of IL-1β, was also associated with fatal outcomes, as identified previously in another study in the first week of hospitalization (22).

Our results revealed that serum levels of the interferon -inducible protein, IP-10, were also associated with a worse prognosis, and it has been suggested that IP-10 may be a good biomarker for predicting disease progression (5, 32). IP-10 exhibits pro-inflammatory and anti-angiogenic properties and has been proposed as a link between inflammation and angiogenesis that affect COVID-19 patients (33, 34). The IP-10 is correlated with IFN type I response. Thus, it makes sense that we found lower concentrations of IFNα in the severe group. Specifically, IFNα is a biomarker mainly produced by virus-infected leukocytes. IFN stimulates the synthesis of major histocompatibility complex class 1 proteins, which are involved in the presentation of viral antigens for recognition by the immune system. The absence of IFN type I expression may impact the ability of dendritic cells to regulate immunity, leading to inadequate priming of antiviral response by CD4 and CD8 T cells (35). SARS-CoV-2 infection triggers proinflammatory response long before IFN-mediated antiviral defenses are induced, if at all. This would explain the long incubation time of the virus and long persistence in the respiratory tract, which may be attributed to delayed and/or reduced production of IFN types I and III (7).

It has been suggested that some cytokine patterns associated with COVID-19 severity, such as M-CSF, IP-10 and IL-1RA, are associated with the presence of the macrophage activation syndrome (36) in which infection of macrophages and dendritic cells potentially plays a major role in COVID-19 pathogenesis, even in the absence of productive infection (28).

Of note, there is a tendency for the values of some biomarkers to be higher in the moderate group than in the severe group (GM-CSF, MCP-1, IL-15, TNFα, IL-1RA, IFNα), which could be due to a delay at the time of hospitalization, due to an overloaded health system in the first wave of COVID-19 in Peru, or due to a delay in taking the sample that favors this tendency.

We found no significant differences in RANTES (CCL5) levels. However, a previous study reported that RANTES was significantly elevated in early stages in moderately but not in severely ill patients (22). We observed a trend to low concentrations in the deceased group and a negative correlation with pro-inflammatory and anti-inflammatory biomarkers, indicating that this biomarker may be related to less severe disease conditions. Apart from cytokines and chemokines, anti-SARS-CoV-2 antibodies play a role in the severity of COVID-19. It has been reported that SARS-CoV-2 antibody levels vary according to sex, blood type, and also severity and mortality (37). The vast majority of patients in this study (87.3%) had detectable anti-S1 IgG levels in the first days after admission. Additionally, higher anti-S1 IgG levels were associated with fatal outcomes. One of the previous studies reporting also higher titers of anti-SARS-CoV-2 antibodies in severe COVID-19 compared to individuals with mild disease also found lower viral loads in severe cases (38). High titers of anti-SARS-CoV-2 IgG in severe patients could activate signaling circuits that dampen cellular responses to interferons by blocking the expression of interferon-stimulated genes associated with milder forms of the disease (38). It has been proposed that this exaggerated response pits the immune system against itself (38). From the point of view of clinical applicability, it is important to highlight that it has been proposed that anti-SARS-CoV-2 IgG can predict improvement better than commonly used clinical parameters such as lymphocyte and neutrophil counts, and ferritin, D-dimer levels (5).

Regarding to the relationship between antibodies and cytokines, anti-spike 1 IgG antibodies presented a positive correlation with EGF and IL-8 and an inverse correlation with IP-10, IFN-γ and G-CSF. A previous study only found a correlation with IL-12p70/IL33 and IgG seroconversion, which correlated with disease severity (39). However, another study hypothesized that IL-8 and overall, the inflammation acts as a mediator between the association of IgG antibodies and the clinical outcome (40).

The limitations of this study include a relatively low sample size (n = 55) and potential false positives as multiple biomarkers and tests were performed without adjusting for multiple testing. Therefore, results should be interpreted with prudence and require further validation but are consistent with published studies in other populations and compatible with the current knowledge of COVID-19 severity, so findings remain relevant. Another limitation is the lack of information about the time period from symptoms onset or positive PCR to admission, which could affect biomarker concentrations. However, our study was carried out in the real-life conditions of COVID-19 cases management at most hospitals in Peru.

Besides stratification of patients at hospitalization, identifying biomarkers associated with mortality in COVID-19 would help to develop new therapeutic approaches and avoid poor outcomes. Immunomodulatory agents could considerably change the expression of these biomarkers. For example, anti-inflammatory drug, dexamethasone has been used in the treatment to COVID-19 as an immunomodulatory agent targeting the IFN-mediated signaling, apart from inflammation (24). At hospital admission, corticosteroids were administered to all patients in Peru and, thus, we could not evaluate its role in the prognosis of COVID-19. Nonetheless, previous studies have reported that its use is not associated with clear benefits in mortality (19), although it has been shown to lower mortality in patients receiving either invasive mechanical ventilation or oxygen alone, but not among those not receiving respiratory support (41). The monoclonal antibody against the IL-6 receptor, tocilizumab, proposed for the treatment of COVID patients was not administered (42) since it was demonstrated not to be effective for preventing intubation or death in moderately ill hospitalized patients with COVID-19 (43).

Finally, it is important to acknowledge that several studies have explored the value of assessing cytokines as predictors of death, finding different results. The various quantification methods, kits, relatively low sample sizes in many studies and populations explored might explain these differences, making evident that further studies should be performed to validate biomarkers.

In conclusion, in this Peruvian cohort, various biomarkers of COVID-19 prognosis and fatal outcome, have been identified, validating previous results in other populations involving the same markers. These results should be further explored to identify novel criteria for COVID-19 patient stratification at hospital admission and potential targets for drug development.

## Data Availability Statement

The raw data supporting the conclusions of this article will be made available by the authors, without undue reservation.

## Ethics Statement

The studies involving human participants were reviewed and approved by Comité de Ética en Investigación para COVID-19 del Seguro Social de Salud – Essalud. Written informed consent for participation was not required for this study in accordance with the national legislation and the institutional requirements.

## Author Contributions

MP, MU, and GM conceived the study. MP, AM-H, BY, and ST performed assay serum antibodies and cytokines, YS and LA participated in the analysis of IgG titters. MU coordinated patient cohort enrolment and clinical data collection. RG and AB participated in sample collection. MP and GM carried out the statistical analyses. MP wrote the manuscript. MU and MP supervised the project. All authors contributed to the article and approved the submitted version.

## Funding

This work was supported by funds from “Kaelin Prize 2020” from EsSalud and internal funds of *Universidad Científica del Sur*.

## Conflict of Interest

The authors declare that the research was conducted in the absence of any commercial or financial relationships that could be construed as a potential conflict of interest.

## Publisher’s Note

All claims expressed in this article are solely those of the authors and do not necessarily represent those of their affiliated organizations, or those of the publisher, the editors and the reviewers. Any product that may be evaluated in this article, or claim that may be made by its manufacturer, is not guaranteed or endorsed by the publisher.
